# The Attitudes of Indian Palliative-care Nurses and Physicians to Pain Control and Palliative Sedation

**DOI:** 10.4103/0973-1075.78447

**Published:** 2011

**Authors:** Joris Gielen, Harmala Gupta, Ambika Rajvanshi, Sushma Bhatnagar, Seema Mishra, Arvind K Chaturvedi, Stef Van den Branden, Bert Broeckaert

**Affiliations:** Interdisciplinary Centre for the Study of Religion and World View (Catholic University Leuven), Sint-Michielsstraat 4 - Bus 3101, 3000 Leuven, Belgium; 1 CanSupport, Kanak Durga Basti Vikas Kendra, Sector 12, R.K. Puram, India; 2 Institute Rotary Cancer Hospital, All India Institute of Medical Sciences, Ansari Nagar, New Delhi, India; 3 Rajiv Gandhi Cancer Institute and Research Centre, Sector 5 Rohini, New Delhi, India

**Keywords:** Attitudes, India, Nurses, Pain control, Palliative care, Palliative sedation, Physicians

## Abstract

**Aim:**

We wanted to assess Indian palliative-care nurses and physicians’ attitudes toward pain control and palliative sedation.

**Materials and Methods:**

From May to September 2008, we interviewed 14 physicians and 13 nurses working in different palliative-care programs in New Delhi, using a semi-structured questionnaire, and following grounded-theory methodology (Glaser and Strauss).

**Results:**

The interviewees did not consider administration of painkillers in large doses an ethical problem, provided the pain killers are properly titrated. Mild palliative sedation was considered acceptable. The interviewees disagreed whether palliative sedation can also be deep and continuous. Arguments mentioned against deep continuous palliative sedation were the conviction that it may cause unacceptable side effects, and impedes basic daily activities and social contacts. A few interviewees said that palliative sedation may hasten death.

**Conclusion:**

Due to fears and doubts regarding deep continuous palliative sedation, it may sometimes be too easily discarded as a treatment option for refractory symptoms.

## INTRODUCTION

When a patient enters the terminal stage of a disease like cancer, intensive treatment of pain and other symptoms will often be required to preserve quality of life. Nevertheless, many medical professionals consider such intensive pain and symptom management controversial. Surveys have shown that large proportions of physicians and nurses still think intensive pain treatment and palliative sedation hasten death. Bendiane *et al* and Peretti-Watel *et al* found that 27% of French home-care nurses and 17% of general practitioners considered prescribing high-dose morphine to be euthanasia.[1,2] In many publications and discussions, euthanasia is understood in a broad sense as a deliberate medical act or omission that has a life-shortening effect that is accepted or aimed at by the physician involved.[3] Therefore, the use of the term euthanasia for the prescription of high-dose morphine by one fifth of French home-care nurses and one sixth of French GPs seems to imply that these groups believe intensive pain control with morphine hastens death. Such beliefs may influence medical decision making at the end of a patient’s life. In a Belgian study, the treating physician thought to have alleviated pain and symptoms with opioid doses that may have shortened the patient’s life in 17.2% of all studied deaths.[4] In the Netherlands, this was the case in 14.7% of all studied deaths.[5] Intensive pain management with opioids is not the only type of pain and symptom management that is often believed to hasten death. Several authors have likened palliative (terminal) sedation to a form of (slow) euthanasia, assisted suicide, or mercy killing in disguise.[6–11] Due to convictions of this kind, pain and other symptoms may remain under treated.[12–14]

In India, fears of addiction and respiratory depression have caused aversion to morphine among medical professionals, and have lead to legal restrictions on the use and distribution of morphine. In recent years, efforts have been made to make morphine more easily available for the treatment of pain in India. Yet, many patients suffering from diseases like cancer and HIV/AIDS still die in pain because availability of morphine remains a problem, the patients, their relatives and the treating physicians are unaware of even the basic principles of palliative care, and the parties involved still fear that intensive pain management may bring about unacceptable side effects, cause addiction to morphine or hasten death.[15–22]

Physicians and nurses who are caring for terminal patients within palliative-care settings in India can be expected to be much more knowledgeable about the complexities of pain control and palliative sedation. Therefore, their attitudes toward pain control and palliative sedation may differ greatly from these of their colleagues working in Indian health care in general. Studies in other countries showed that notions and opinions about pain management are influenced by experience in terminal care. Hollen *et al* observed that practice setting and pain education influence knowledge, as well as attitudes, about pain.[23] The above-mentioned French study of Bendiane *et al* showed that experience in terminal care influences attitudes to pain management. The authors of this study found that the opinion that prescribing high-dose morphine to terminally ill patients is euthanasia is more frequently held by nurses who have not followed terminally ill patients during the previous three years, and by nurses with less knowledge about pain management involving opioid analgesics.[1]

From these observations, we may not assume, however, that Indian palliative-care nurses and physicians consider pain control and palliative sedation unproblematic. Expert knowledge may make them more aware of more profound ethical issues and context specific problems related to pain control and palliative sedation. So far, the attitudes of Indian palliative-care nurses and physicians toward pain control and palliative sedation have not been studied. The aim of the present study is to assess Indian palliative-care nurses and physicians’ opinions and attitudes toward pain control and palliative sedation, and to find out to what extent Indian palliative-care physicians and nurses consider pain control and palliative sedation acceptable and for which reasons. In this study, pain control is understood as the intentional administration of analgesics and/or other drugs in dosages and combinations required to relieve pain adequately. Palliative sedation is defined as the intentional administration of sedative drugs in dosages and combinations required to reduce the consciousness of a terminal patient as much as necessary to adequately relieve one or more refractory symptoms.[3,24,25]

## MATERIALS AND METHODS

Since Indian palliative-care physicians and nurses are experienced in the treatment of pain and symptoms, their attitudes toward pain control and palliative sedation can be diverse and complex. Therefore, an explorative qualitative research design was required to gain insight into these attitudes. We opted for face-to-face interviews following grounded-theory methodology[26,27] using a semi-structured questionnaire. To gain access to eligible participants, we contacted the directors of four institutions and organizations that provide palliative care in New Delhi and explained them the aims and methodology of the research project. The directors of all contacted institutions and organizations were willing to support the research project and granted permission to interview physicians and nurses working in the palliative-care programs. In two institutions, there was an ethics committee, and approval of the research project by these committees was required before any physician or nurse working in these institutions could be interviewed. The directors of the palliative-care programs in these institutions obtained the approval of the respective ethics committees. The directors of the palliative-care programs provided a list with the names of physicians and nurses in their institutions or organizations who had been working in Indian palliative care since at least 5 months, and who were fluent in either Hindi or English. The number of interviewees was not fixed in advance, but depended on when theoretical saturation would be reached. Theoretical saturation occurs when further data collection and analysis would not yield new aspects or insights. Theoretical saturation was reached after 27 interviews.

The semi-structured questionnaire which was used during the interviews covered several issues related to palliative care, ethical attitudes, and worldview. We asked the interviewees to provide some demographic information about themselves and to explain why they were working in palliative care. The questionnaire contained several questions about the respondents’ experiences with patients. The participants were offered eight hypothetical cases dealing with treatment decisions in advanced disease. Two of these cases dealt with pain control and palliative sedation. We also extensively enquired after the interviewees’ ideological and religious views and practices. The order of the questions and cases in the interviews was not fixed. As much as possible questions were asked and cases presented in relation to what the interviewee had said. Answers of the interviewees to questions or cases triggered new questions, which were sometimes also asked in subsequent interviews to obtain a clearer picture of the opinions and attitudes of Indian palliative-care physicians and nurses. In the present article, we will only deal with the interviewees’ reactions to the cases dealing with pain control and palliative sedation. The other issues that were dealt with during the interviews were part of other separate studies and are discussed in separate articles.

We solicited the interviewees’ opinions on pain control and palliative sedation through two hypothetical cases each followed by a focused question. In the first case, a patient’s pain was controlled with a high dose of painkillers and the interviewee was asked whether there is any limit for the administration of these painkillers. In the second case, a patient was experiencing uncontrollable pain and the treating physician decided to use palliative sedation. The interviewee was asked whether palliative sedation is a proper treatment in this case. To avoid confusion over the meaning of the term palliative sedation, the term palliative sedation was not used in the case that was read to the participants. In order to allow interviewees to make ethical distinctions by themselves, initially several aspects of the treatment decisions were purposefully left undetermined in both cases.

### Case 1 (pain control)

A terminal patient is in severe pain. Is there any limit for the administration of painkillers?

### Case 2 (palliative sedation)

A terminal patient is in severe pain. The physician is unable to treat the pain while the patient is in a conscious condition. Therefore, he decides to reduce the consciousness of the patient with sedative drugs to adequately relieve the pain. Is this a proper treatment?

Fourteen physicians and 13 nurses were interviewed from May to September 2008. The interviews were conducted at a quiet location at the interviewee’s workplace. Before the formal start of the interview, the interviewer (Joris Gielen) explained the general aim of the interview and the research project to the interviewee. The interviewer promised the interviewee that only the interviewer would have full access to the nonanonymous data, and that the names of the participants would not be mentioned in any reports about the study. Since physicians and nurses working in palliative care in New Delhi come from different parts of India, the interviewees were informed they could express their ideas and opinions in either Hindi or English, the two languages that are most common in New Delhi and are also understood and spoken by the interviewer. The interviewer also asked permission to record the interview as this would allow him to transcribe the interview word by word, which would, in turn, enhance the reliability of the collected data. Two respondents (one physician and one nurse) said they did not feel comfortable with the idea that the interview would be recorded. In these cases, the interview was not recorded and the interviewer made extensive written notes which were typed out and completed by the interviewer shortly after the interview. All other interviews were recorded. On average, each interview took 50 min.

Data collection and analysis progressed simultaneously. Every recorded interview was transcribed verbatim by the interviewer. The data were analyzed by the interviewer following Glaser and Strauss’ approach of grounded theory. Key concepts were identified in the interviews. Codes were added to the data. Through constant comparisons, categories were determined and associations between categories were clarified. For the data analysis, MAXQDA 2007 was used. The reliability of the data and analysis was enhanced by applying theoretical sampling and including evaluative feedback from the research supervisors. Theoretical sampling implies that researchers strive for a sample that is as heterogeneous as possible in order to maximize the possibility of discovering new data that could elucidate the evolving theory. While the data collection and analysis were in progress, regularly the outcomes of the research were discussed with the supervisors. The supervisors commented on the results and offered suggestions for the analysis and interpretation of the data.

To protect the anonymity of the participants, their names have not been mentioned in the text in the results section. The name of each respondent has been replaced by a number. Each number has been assigned to one particular participant. Whenever reference is made to a particular participant, the unique number that refers to him or her has been added between brackets.

## RESULTS

### Demographic characteristics

Seventeen participants were female and 10 were male. The average age of the interviewees was 42.7 years (S.D. 11.09). Nine participants were born in Kerala, 5 in Uttar Pradesh, 3 in Haryana, 2 in Assam, 2 in New Delhi, 1 in Tamil Nadu, 1 in Rajasthan, 1 in Punjab, 1 in Maharashtra, 1 in Uttarakhand, and 1 in Madhya Pradesh. Eleven participants were working in palliative home care, 4 in a hospice, 3 in a palliative-care unit in a hospital, 2 in a pain clinic, and 7 in a pain clinic and a palliative-care unit. Eight participants were Christian, and 19 were Hindu. All participants were fluent in either Hindi, English, or both.

### Attitudes to pain control

Although the palliative-care nurses and physicians are aware many people in India think painkillers hasten death, they themselves think otherwise. The physicians and nurses are convinced painkillers do not hasten death if properly titrated. They are convinced pain can most often be controlled without causing unacceptable side effects.

In their practice, the nurses and physicians experience that the Indian general public and many health professionals still believe that pain killers and particularly morphine hasten death. A home-care nurse (5) told that before joining a palliative home-care team she also was afraid of administering morphine. A home-care physician (3) testified that patients and their relatives are often afraid of morphine.

“Patients have this misbelief. They think that they are taking morphine and because of that they will die faster”.

A home-care nurse (2) told that due to the conviction that painkillers hasten death the palliative-care physicians and nurses are sometimes wrongfully accused of hastening death while administering pain treatment. She recalled that she was called once to the house of a patient who was experiencing unbearable pain. With the palliative-care physician’s permission, the nurse administered an injection to control the pain. After 20 min the patient passed away. The relatives accused the nurse of having administered a lethal injection.

The Indian palliative-care physicians and nurses do not share this fear of painkillers and morphine with the Indian general public and other health-care professionals. Among the interviewees, there is hardly any fear that the administration of painkillers in large doses will hasten death or cause unacceptable addiction. In the opinion of the participants, death will not be hastened and respiratory depression will not occur provided painkillers are properly titrated. A home-care physician (4) expressed this idea very clearly.

“Painkillers do not hasten death. If given rationally, properly titrated they do not hasten death. If properly titrated! This is important”.

The interviewees stressed the importance of proper titration of painkillers. The drugs have to be titrated against pain, while taking possible side effects into consideration. A physician who works in a pain clinic (9) explained: “The drug which is most commonly used to treat severe pain is morphine. In addition, as long as the patient is having pain, he will not get sedated or he will not have that respiratory depression. This means, whatever serious complications, as long as the pain is not controlled, these side effects will not appear”.

According to the physicians and nurses, the patient’s pain is the single most important criterion while treating pain. They argue that there is no absolute upper limit for the administration of painkillers. A physician practicing in a palliative-care unit and a pain clinic (13) testified: “Morphine can give side effect. However, as far the drug is concerned, morphine is the only drug that does not have an upper dosing limit. It is the only drug in the world that does not have any ceiling effect. If a person is in pain, we can increase the morphine dose according to the pain. It has no upper limit”.

According to the physicians and nurses, pain has to be controlled and there are many ways to control pain effectively without hastening death. A home-care nurse (5) said: “There is no limit [for the administration of pain killers]. If a particular drug does not work, then we can give another. If that drug also does not work, we can administer an injection. We can also put [nerve] blocks. As such there is no limit. […] The drugs can have side effects. However, no one will die due to the drugs”.

The palliative-care physicians do not ignore possible negative side effects like addiction and respiratory depression. However, in their opinion fear of these side effects should not impede pain-management. The interviewees are of the opinion that in most cases pain can be effectively controlled without causing unacceptable side effects. A physician working in a pain clinic (9) warned against addiction, yet added that the possibility of addiction should not be an obstacle to adequate pain management.

“The only concern is about this addiction, which can occur. However, just because the patient will get addicted, we should not prevent the pain control. We should keep on giving whatever dose for pain control. Even if the patient gets addicted to it, the control of pain – I think - is more important than this”.

A hospice nurse (22) said that while treating pain the palliative-care team should beware of respiratory depression. Yet, in all situations, there are ways to relieve pain without causing respiratory depression.

“We start with a small dose of morphine first. And then as the patient tolerates we increase. In addition, when the patient is having pain, then other supportive pain killers will be given along with that. However, if the patient is having any respiratory distress with the morphine, then we will stop. We do not continue. We stop morphine then. Not all the patients are having a problem with morphine, maybe one or two. Then we stop. We do not continue that. Then we take another alternative”.

For almost all interviewees the administration of painkillers in doses which are not carefully titrated against the patient’s pain is both unnecessary and ethically unacceptable. Only two physicians (6, 7) were of the opinion that in the rare case of a terminal patient with very limited life expectancy, who is in severe uncontrollable pain, the limits of proper titration could be transgressed even if such treatment might hasten the patient’s death. In this context, one of these physicians (6) spoke about the administration of a possibly toxic dose of pain killers.

“In these situations, we can give narcotics, analgesics to the point of using maybe toxic doses of the drug to alleviate the pain”.

### Attitudes to palliative sedation

Some interviewees argued that reduction of consciousness is not indicated to control physical pain. According to them, consciousness should rather be reduced to control anxiety and apprehensiveness. The physicians and nurses considered mild palliative sedation acceptable. A majority of the interviewees were unfavorably disposed to deep continuous palliative sedation. These interviewees argued such sedation may cause unacceptable side effects, that it impedes basic daily activities and social contacts, and that pain can be controlled sufficiently without it. Several interviewees were of the opinion that palliative sedation may hasten death, but this assumed death-hastening effect of palliative sedation was not used to argue against palliative sedation. Those who considered deep continuous palliative sedation acceptable argued the patient’s needs and life expectancy should be carefully assessed before such sedation could be administered.

After hearing the case about palliative sedation, some interviewees questioned the relevance and accuracy of the case. They argued that reduction of consciousness is not a proper treatment to control physical pain. According to these interviewees, physical pain should be treated separately with pain killers. In their opinion, sedatives should rather be administered when a patient is very anxious or apprehensive. Two physicians who were working in a palliative care unit and a pain clinic in a hospital (16, 27) added that it is pointless to try to manage physical pain by sedating the patient, because according to them a sedated patient can still experience physical pain. One of these physicians (27) expressed this idea in the following way.

“See, why do you want to do the sedation? Tell me that first. I mean, you want to tell the patient that he should not cry in pain because he or she is suffering from pain. The main point is that if he or she is suffering from it, then give him or her analgesia. What is the point of giving sedation? You are just calming the patient so that he will sleep, but he will suffer due to pain”.

Remarks of this kind notwithstanding, mild palliative sedation was considered acceptable by the interviewees. Some interviewees based their approval of mild palliative sedation on their experience that initially some kind of drowsiness or reduction of consciousness can also occur while administering analgesics like morphine. These participants did not make a clear distinction between mild palliative sedation, i.e., a light reduction of consciousness which is intentionally induced with sedative drugs to relieve one or more refractory symptoms, and a sedated state which occurs as an unintended side effect of the administration of analgesics. The confusion between mild palliative sedation and a light sedation unintentionally caused by analgesics seems to be partially rooted in the conviction that morphine can be used as a sedative agent. A few participants thought that morphine can be administered to sedate a patient with the aim of relieving refractory symptoms.

The physicians and nurses disagreed whether palliative sedation should always remain light or can also be deep and continuous. Most physicians and nurses who discussed the issue of deep continuous palliative sedation did not favor this practice. Nurses and physicians who were against deep continuous palliative sedation were of the opinion that reduction of consciousness should always be light and sedatives are preferably only administered at night to help the patient to sleep well. A home-care nurse (2) and a physician working in a pain clinic and a palliative-care unit (12) pointed out that continuous deep palliative sedation may cause unacceptable side effects, like problems with respiration, urination and bowel movement. Some interviewees were against continuous deep palliative sedation because such sedation impedes basic daily activities and social contacts. An anesthetist working in a pain clinic (9) expressed this idea in the following way.

“The idea is to control pain and the patient should be able to live his active life, even if he is having metastases. So just by putting him to sleep all the time he is not leading an active life. Whatever is required is that he should live his normal life”.

A nurse who works in a palliative-care unit (18) told that a patient should be able to communicate because otherwise the caregivers will not know whether the patient is comfortable.

“According to me, there is no advantage [in deep continuous sedation]. The patient cannot talk to his relatives. How will his relatives come to know his condition or pain? What can they do [for him]? They will not know anything”.

The physicians and nurses are aware that at the last stage of life the relatives want to communicate with their dying family member, and therefore the family members do not want that the patient remains in a deeply sedated condition until death. A physician working in a pain clinic and a palliative-care unit (8) explained: “The relatives know that the patient is at the last stages of his life. However, they want the patient to remain conscious because they want to interact with the patient at the last moments. That is why they do not want the patient to be made unconscious”.

A few interviewees stressed the importance of oral nutrition and hydration. A patient who is in deep continuous sedation can only receive artificial nutrition and hydration, which may also cause complications. A few participants were not in favor of continuous deep palliative sedation for the treatment of pain because in their opinion pain can be controlled sufficiently without continuous deep palliative sedation.

Although several interviewees had said that palliative sedation may hasten death, no one mentioned the assumed death-hastening effect of palliative sedation as an argument against palliative sedation. Most likely, the interviewees did not mention this argument because they were not absolutely sure that palliative sedation indeed hastens death. An anesthetist working in a palliative-care unit and a pain clinic (7) expressed his doubts in the following way.

“We do not overdose the patient, but maybe it is [hastening his death]. We actually do not know whether it is because of the drug or because of the disease itself. Some diseases progress rapidly. We prescribe sedatives to relieve the patient’s pain”.

Some interviewees explicitly said they were not absolutely averse to deep continuous palliative sedation. These palliative-care professionals advocated a careful assessment of the patient’s needs and life expectancy before administering deep continuous palliative sedation. An anesthetist working in a palliative-care unit and pain clinic (8) made the following statement about patients who are eligible for continuous deep palliative sedation.

“See, we cannot define, or we cannot determine a rule of thumb or a set of guidelines for managing these patients. It is basically a case to case basis. In addition, we have to modulate. We have to change our plans or our treatment part according to the patient’s need”.

## DISCUSSION

The Indian palliative-care nurses and physicians believe that in most cases pain can be effectively controlled. They argue that medical advancements in the treatment of pain almost always allow the palliative-care team to ease physical pain to acceptable levels. They are also convinced that intensive pain control does not hasten death, provided painkillers are correctly administered and properly titrated. Regarding pain control, the opinions of the Indian palliative-care physicians and nurses are in line with the evidence of studies showing that painkillers even in high doses do not hasten death if administered properly and titrated according to pain.[28–35] Since Indian palliative-care nurses and physicians think properly titrated painkillers do not hasten death, they do not consider administration of pain killers in large doses an ethical problem.

The observation that palliative-care physicians and nurses do not consider administration of painkillers in large doses an ethical problem should be taken into consideration when conducting further research on the attitudes of medical professionals toward pain control. The authors of many earlier quantitative studies on physicians or nurses’ attitudes and practices regarding pain control have assumed that intensive pain control does hasten death. The assumption of the death-hastening effect of intensive pain control has influenced the wording of the questions regarding pain control in these surveys. This has, for instance, been the case in the Dutch surveys conducted since the early 1990s by van der Maas *et al*, in which the contacted physicians were asked whether, “to alleviate pain or symptoms, they had ever decided to give such dosages of opioids that they had to accept the risk of shortening the patient’s life.”[5,36] Also in more recent surveys similar questions can be found. Van der Heide *et al* asked physicians whether they had intensified “the alleviation of pain and suffering while taking into account the possibility or certainty that this would hasten the patient’s death, or partly with the intention of hastening the patient’s death”.[37] Parker *et al* asked their respondents whether they would be ready to intensify the alleviation of symptoms by using drugs in a patient who is experiencing severe pain or suffering, “taking into account the probability or certainty that this could hasten the end of a patient’s life.”[38] Such questions are not in line with recent findings that show that proper pain control does not hasten death, and may confuse respondents like the Indian palliative-care nurses and physicians, who do not share the opinion that administration of painkillers in large doses hastens death.

The physicians and nurses in this study are not absolutely opposed to palliative sedation. Yet, they warn that palliative sedation should be administered carefully and for the right refractory symptoms. Some interviewees pointed out that reduction of consciousness is not a proper treatment to control physical pain. They argued that palliative sedation should be used to control refractory psychological suffering or anxiety. The focus on psychological distress as a refractory symptom that may require palliative sedation may sound remarkable since in earlier surveys medical professionals considered refractory physical suffering the most important indication for palliative sedation. In Connecticut, 78% of internists found palliative sedation ethically appropriate if a terminally ill patient has intractable pain despite aggressive analgesia.[39] Two other studies showed that physicians and experts in medical ethics are more likely to approve of palliative sedation in the case of a patient who is suffering physically, rather than in the case of a patient who is going through existential or mental suffering.[10,40] Yet, Muller-Bush *et al* observed that from 1995 to 2002 the main indication for palliative sedation had shifted from refractory physical symptoms to psychological distress.[41] In the West, this shift has been explained by advancements in the treatment of physical suffering. Due to these advancements patients are less troubled by physical suffering, and focus more on existential or psychological issues.[42,43] The increased focus on existential or psychological issues by the patients may have made the treatment of refractory psychological distress more urgent. In India, a significant proportion of palliative-care physicians and nurses seem convinced that pain can always be sufficiently controlled without palliative sedation, and that, therefore, palliative sedation should not be used to treat physical pain.

As long as the reduction of consciousness remains light, the physicians and nurses in this study do not consider palliative sedation problematic. A few interviewees likened mild palliative sedation to the light reduction of consciousness which can occur as an unintended side effect of the administration of analgesics. These participants did not make an ethical distinction between these two types of sedation. In the case of palliative sedation, sedation is purposely induced to control refractory symptoms. In the other case, the reduction of consciousness is an unintended side effect of pain control with analgesics. For a few participants, confusion of these two types of sedation seems to be rooted in the idea that morphine can be used to reduce a patient’s consciousness with the aim of relieving refractory symptoms. Research has shown, however, that morphine is not a suitable drug for that purpose.[44]

For various reasons, many participants consider deep continuous palliative sedation a questionable practice. The concerns voiced by the palliative-care nurses and physicians regarding palliative sedation in general and deep continuous palliative sedation in particular show that the interviewees are committed to the physical, psychological and social wellbeing of the patients and their relatives, and that they do not opt for palliative sedation as an easy way out. Yet, not all of the nurses and physicians’ concerns may be justified, and might in some cases even prevent the physicians and nurses from administering deep continuous palliative sedation to patients who may actually be better off with deep continuous palliative sedation. Two opinions expressed by the interviewees regarding deep continuous palliative sedation are disputable: the opinion that palliative sedation especially when deep and continuous, may hasten death, and the opinion that deep continuous palliative sedation will lead to unacceptable social loss.

The conviction that palliative sedation may hasten death is held by some physicians experienced in terminal care. In the study of Morita *et al*, 17% of Japanese oncologists and palliative-care physicians considered palliative sedation practically indistinguishable from acts to hasten death.[45] Yet, most studies comparing survival of sedated and non-sedated patients have not found significant differences in survival time.[34,46–50] Sykes and Thorns even observed that patients who had received sedation during the last seven days of their lives had longer survival from admission than patients who received no sedation or those who received sedation only in the last 48 hours of life.[51] The Indian palliative-care nurses and physicians may also have noticed that the differences in survival time between sedated and nonsedated patients are limited, if at all occurring. This may explain their reluctance to use the assumed death-hastening effect of palliative sedation as an argument against the treatment.

Some interviewees objected to continuous deep palliative sedation because such sedation would impede basic daily activities and social contacts. The interviewees argued that a patient who remains deeply sedated until death is unable to eat and drink orally, and cannot converse with relatives and friends. Although communication with family and friends, and the oral intake of food and liquid are important for the patient and his or her relatives until the very last moment of a patient’s life, these activities should not be used as an absolute criterion to judge of the ethical acceptability of a treatment like palliative sedation. They are just two of the aspects that have to be taken into consideration while deciding on the administration of palliative sedation. In studies, the mean survival after the onset of palliative sedation has been found to be just one to six days.[42] The palliative-care team, the patient, and his or her relatives should decide together whether the possibility to communicate and feed the patient orally during these few days outweighs the benefits brought by deep continuous palliative sedation.

## CONCLUSION

This study shows that Indian palliative-care nurses and physicians do not consider the administration of painkillers in large doses an ethical problem, provided the pain killers are administered correctly and titrated against the patient’s pain. Although the physicians and nurses consider light reduction of consciousness acceptable, some interviewees have doubts and fears regarding deep continuous palliative sedation. Due to these fears and doubts, deep continuous palliative sedation may sometimes be too easily discarded as a treatment option for refractory symptoms.
